# Dihydromyricetin prevents cardiotoxicity and enhances anticancer activity induced by adriamycin

**DOI:** 10.18632/oncotarget.2410

**Published:** 2014-09-05

**Authors:** Hong Zhu, Peihua Luo, Yingying Fu, Jincheng Wang, Jiabin Dai, Jinjin Shao, Xiaochun Yang, Linlin Chang, Qinjie Weng, Bo Yang, Qiaojun He

**Affiliations:** Zhejiang Province Key Laboratory of Anti-Cancer Drug Research, Institute of Pharmacology & Toxicology, College of Pharmaceutical Sciences, Zhejiang University, Hangzhou 310058, China

**Keywords:** Adriamycin, cardiotoxicity, Dihydromyricetin, ARC, anticancer

## Abstract

Adriamycin, a widely used anthracycline antibiotic in multiple chemotherapy regimens, has been challenged by the cardiotoxicity leading to fatal congestive heart failure in the worst condition. The present study demonstrated that Dihydromyricetin, a natural product extracted from ampelopsis grossedentat, exerted cardioprotective effect against the injury in Adriamycin-administrated ICR mice. Dihydromyricetin decreased ALT, LDH and CKMB levels in mice serum, causing a significant reduction in the toxic death triggered by Adriamycin. The protective effects were also indicated by the alleviation of abnormal electrocardiographic changes, the abrogation of proliferation arrest and apoptotic cell death in primary myocardial cells. Further study revealed that Dihydromyricetin-rescued loss of anti-apoptosis protein ARC provoked by Adriamycin was involved in the cardioprotection. Intriguingly, the anticancer activity of Adriamycin was not compromised upon the combination with Dihydromyricetin, as demonstrated by the enhanced anticancer effect achieved by Adriamycin plus Dihydromyricetin in human leukemia U937 cells and xenograft models, in a p53-dependent manner. These results collectively promised the potential value of Dihydromyricetin as a rational cardioprotective agent of Adriamycin, by protecting myocardial cells from apoptosis, while potentiating anticancer activities of Adriamycin, thus further increasing the therapeutic window of the latter one.

## INTRODUCTION

Adriamycin (ADR), which belongs to anthracyclines, is one of the most widely prescribed and effective cytotoxic drugs used in oncology, involved in the treatment of many tumor types and associated with favorable clinical outcomes, including multiple myeloma, neuroblastoma, leukemia, sarcoma, lymphoma and so on [1, 2]. However, coming along with that, a serious adverse effect being life-threatening heart damage tempestuously limited its therapeutic potential [3, 4]. Increasing studies showed that myocardial toxicity manifested in its most severe form by fatal congestive heart failure (CHF) may occur either during the ADR therapy or months to years after the termination of the therapy, and the toxicity wound radically increased with the accumulation of ADR in a dose-dependent manner [5]. During the retrospective analysis conducted in America in 2002, the risk of development of CHF would reach up to 26% when the cumulative doses came to 550 mg/m^2^, and the fatality rate would be 30%-50% approximately [6]. In order to minimize ADR associated cardiotoxicity, several approaches could be considered: the rigorous cardiac monitoring, the use of anthracycline analogs with lower cardiotoxicity, and modifications of the program of administration [7]. Among them, the introduction of cardioprotective agents has been paid extensive attention.

In the last 40 years after the first uncovering of the serious cardiotoxicity caused by ADR, numerous efforts have been devoted to explore the effective strategies to ameliorate it. Several agents including amifostine [8], phenylbutyrate [9], and glutathione [10], were found to alleviate ADR-induced cardiotoxicity. However, only one compound, dexrazoxane, discovered by Kurt Hellmann in 1972, is used as a cardioprotective agent in clinical [11, 12]. The mechanistic studies revealed that dexrazoxane could act as a derivative of EDTA, chelate iron and thus reduce the superoxide radicals to decrease the apoptosis of myocardial cells [13, 14]. However, several lines of evidences from recent clinical trials implicated the interference with anti-cancer activity of ADR, as well as the higher latent risk for acute myeloid leukemia syndrome and other secondary malignancies in pediatric patients when dexrazoxane was applied [15, 16]. These findings prompt that it is far from enough in discovering more safe and clinical effective protectors. Thus the major challenge, accompanying with the great interest in this field relies on the discovery of novel cardioprotective agent(s) which could promote a selective reduction of heart damage with more safety, and importantly, without decreasing the anticancer efficacy, thus achieve the optimal clinical use of ADR.

Being served as a major source of drugs for centuries, natural products with a broad spectrum of applications were highly expected in finding less-toxicity but high-efficiency pharmaceuticals. Dihydromyricetin (DMY), also known as ampelopsin, is a type of flavonoid extracted from ampelopsis grossedentata. The compound is credited with hepatoprotective effects and antioxidant activity in anterior researches [17, 18]. In the present study, we uncovered the cardioprotective activity of DMY in ADR-treated primary myocardial cells, H9C2 cells and mice models. Further study suggested that DMY could accumulate ARC (apoptosis repressor with caspase recruitment domain), an important anti-apoptotic factor in myocardial cells, through modulating the E3 ubiquitin ligase MDM2. Intriguingly, DMY exhibited a synergistic effect with ADR in its anticancer activity in a p53-dependent manner both *in vitro* and *in vivo*. Taken together, this study represents the first attempt to characterize the dual-mode of DMY on the ADR-driven cardiotoxicity and anticancer capacity, which highlights the promising potential of DMY as a cardioprotective agent in the clinical use of ADR.

## RESULTS

### Dihydromyricetin protects against adriamycin-induced cardiotoxicity *in vivo*

**Figure 1:**
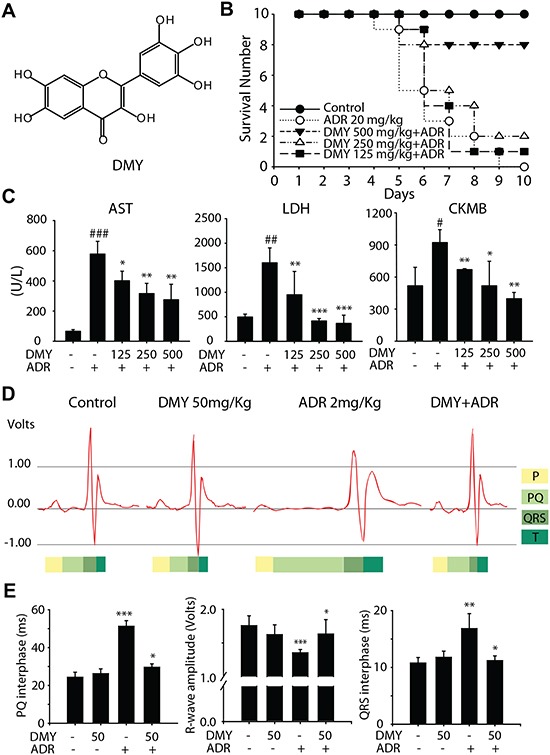
Dihydromyricetin decreaseded Adriamycin-induced cardiotoxicities *in vivo* **(A)** The chemical structure of DMY. **(B)** The survival experiment of ICR mice. The ICR mice were pretreated with DMY, followed by a single dose of ADR (20 mg/kg, ip) as described in Materials and Methods. The survival number was counted each day: control (n = 10), ADR (n = 10), ADR + DMY 125 mg/kg (n = 10), ADR + DMY 250 mg/kg (n = 10), ADR + DMY 500 mg/kg (n = 10). **(C)** The changes in serum cardiac enzyme activity of AST, LDH and CKMB in drug-administered ICR mice. As described above serum cardiac enzyme activity of AST, LDH and CKMB were detected in the sixth day by full-automatic biochemical detect machine using specific detective kits. Data are expressed as mean ± SD (n=4). **(D and E)** The protective effect of DMY on ADR-driven abnormality of electrocardiogram. The electrocardiogram was detected according to the Materials and Methods in non-drug treament group, 50 mg/kg DMY treatment group, 2 mg/kg ADR treatment group and co-administered group (n=4).

As a natural product, DMY is an extract isolated from ampelopsis grossedentata, and its chemical structure was showed in Figure 1A. In order to test the protective effect of DMY in ADR-induced cardiotoxicity, the imprinting control region (ICR) mice survival experiment was performed. As shown in Figure 1B, the mice were first died on day 5 after a single administration of ADR at the dose of 20 mg/kg, and the survival rate was 30% on day 7. In the contrast, when combined with DMY at the dosage of 500 mg/kg, 250 mg/kg, 125 mg/kg, the survival ratio increased to 80%, 50% and 40% respectively (day 7). Of note is the fact that, although all the mice in ADR treatment group were died out in the next three days, the survival rate of 500 mg/kg DMY treated group was still maintained at 80%. During the survival experiment, we also monitored the serum cardiac enzyme activity of aspartate aminotransferase (AST), lactate dehydrogenase (LDH) and creatine kinase MB (CKMB). As expected, the ADR treated group could induce a significant increase in serum cardiac enzyme activity of AST, LDH and CKMB, comparing to the control group. These enhanced levels of cardiac enzyme activity would be significantly ameliorated by DMY cotreatment in a dose-dependent manner (Figure 1C). In addition, the similar effects on these cardiac enzyme activity of DMY were also detected in the cell supernatant of cultured primary myocardial cells (Figure S1). These results indicated that DMY exhibited cardio-protective activities against the acute cardiac injury caused by ADR. Furthermore, we observed the effect of continuous administration of DMY for 25 days (50 mg/kg) on ADR (2 mg/kg)-driven abnormality of electrocardiogram using ICR mice as models, to evaluate the protection under a long-term condition. For this prolonged experiment, the dosages of both compounds were scaled down to prevent the animal death. The protective effect of DMY on ADR-driven abnormality of electrocardiogram was also observed. As shown in Figure 1D and 1E, pretreatment with DMY could reduce ADR conducted prolonged P-R interval, QRS duration and decreased amplitude of R wave. Collectively, these data clearly demonstrated that DMY could protect against ADR-induced cardiotoxicity.

### Dihydromyricetin attenuates the adriamycin-induced rat primary cardiomyocytes apoptosis and reactive oxygen species

**Figure 2:**
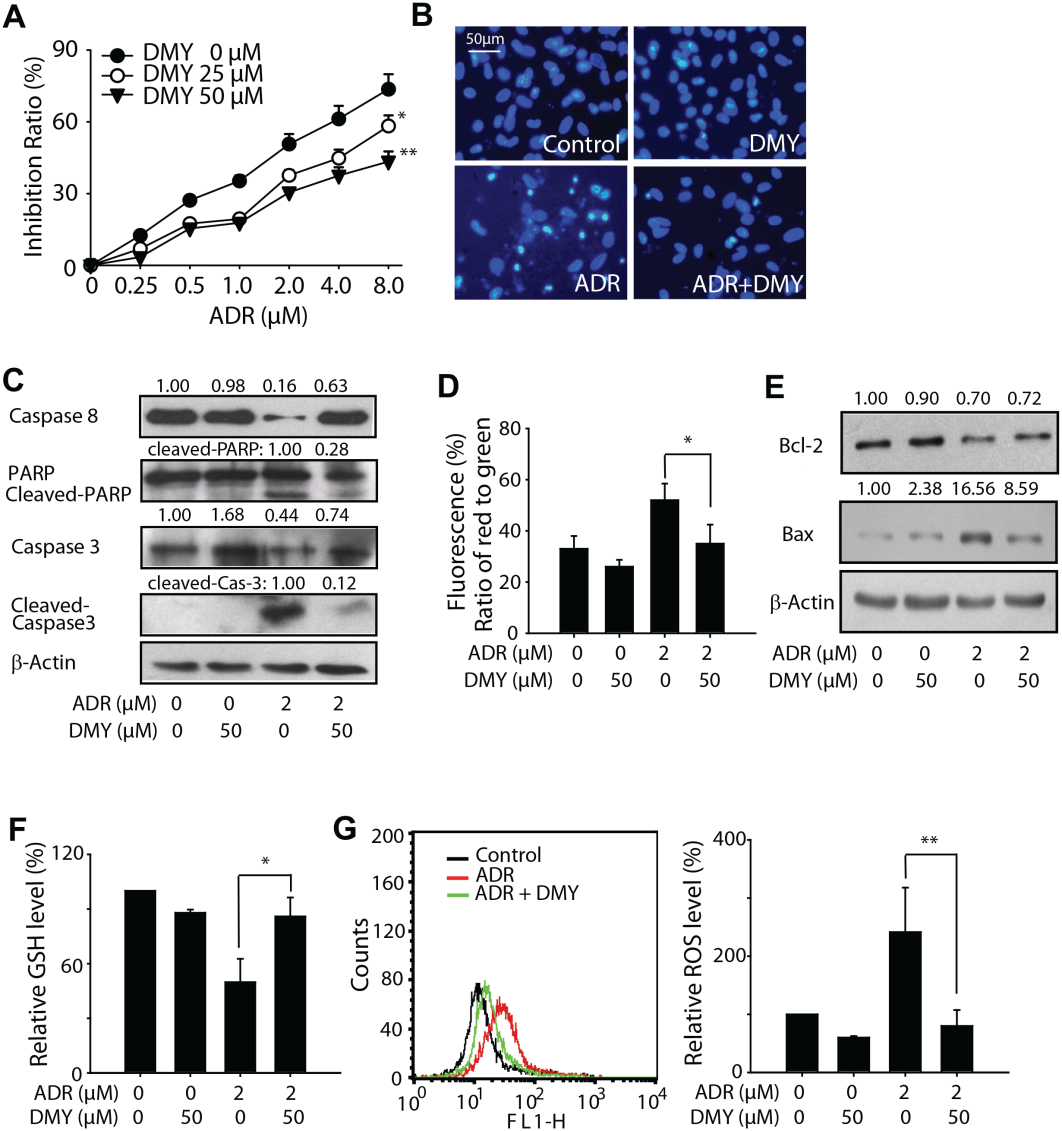
Dihydromyricetin reduced primary myocardial cell apoptosis and reactive oxygen species caused by Adriamycin **(A)** Effects of DMY on ADR induced proliferation inhibition of cultured neonatal rat heart myocytes. Cells were seeded at a density of 5×10^3^ per well on 96 well plate and treated with DMY (DMY was pretreated 24 h earlier than ADR) and ADR as the indicated concentrations, inhibition rate was valued by MTT assay 24 h later after ADR treatment (n=4). **(B and C)** Effects of DMY on ADR-induced apoptosis in cultured neonatal rat heart myocytes. Cells were treated with or without 50 μM DMY for 24 h, followed by incubated with 2 μM ADR for another 48 h, nuclei changes were photographed with fluorescence microscope (B); whole cell lysates were analyzed by western blot, caspase-3, caspase-8, PARP and their cleaved fragments were analyzed (C). **(D)** Effects of DMY on loss of mitochondrial membrane potential in cultured neonatal rat heart myocytes. After treatment with DMY and ADR as the indicated concentrations, a loss of mitochondrial membrane potential was detected by flow cytometry after JC-1 staining. The green fluorescence intensity indicated the cells with low mitochondrial membrane potential, while the red fluorescence intensity indicated the cells with stable mitochondrial membrane potential (n=4). **(E)** Effects of DMY on Bcl-2 and Bax protein expression induced by ADR in cultured neonatal rat heart myocytes. After 24 h of treatment with or without 50 μM DMY, cells were incubated with 2 μM ADR for 48 h and lysed, Bcl-2 and Bax were analyzed by western blot. **(F and G)** Effects of DMY on ADR induced ROS in cultured neonatal rat heart myocytes. Cells were seeded into 24-well plates (5×10^4^/ml) and incubated with indicated drug, GSH level was measured by a spectrophotometer at 405 nm with the sample prepared according to assay kit 24 h later after drug treatment (n=4) (F), the ROS level was measured using the oxidation sensitive fluorescent dye Carboxy-DCFDA by fluorescence spectrometer 3 h later after drug treatment (n=4) (G).

Since numerous studies have demonstrated that the cardiotoxicity caused by ADR is attributed to multiple mechanisms eventually leading to myocardial cells apoptosis [22], we were next encouraged to examine the effect of DMY on ADR-induced rat primary myocardial apoptosis. By applying the neonatal rat cardiomyocytes, we found that the cell proliferation was inhibited after treating with ADR in a concentration-dependent manner. On the contrary, DMY treatment significantly abated the inhibition effects induced by ADR (Figure 2A). To validate the apoptosis induction caused by ADR, both the DAPI staining and Western blot analysis were performed. As shown in Figure 2B, ADR treatment resulted in extensive nuclear condensation and fragmentation in neonatal rat cardiomyocytes, while pretreatment of DMY for 24 h developed a decrease in nuclear abnormity. Western blot analysis revealed an increase of caspase-3, caspase-8 and PARP cleavage fragment expression caused by ADR treatment could be blocked by the administration of DMY (Figure 2C). As the loss of mitochondrial membrane potential is one of the most remarkable events in early apoptosis, JC-1 staining combined with flow cytometry was conducted, and the results showed that DMY preprocessing could antagonize the loss of mitochondrial membrane potential caused by ADR (Figure 2D). In addition, we also observed that the anti-apoptotic factor Bcl-2 was up-regulated and the pro-apoptotic factor Bax was down-regulated in the DMY+ADR group comparing to ADR group (Figure 2E). Taken together, these results further demonstrated that DMY protected the cells from ADR-induced mitochondrial-associated apoptosis.

Given that (i) several clues of evidence suggest the critical role of reactive oxygen species (ROS) in ADR-induced cardiotoxicity [23, 24], (ii) the presence of phenolic hydroxyl groups in DMY implies its potential antioxidant activity (Figure 1A), we next measured the changes of intracellular GSH and the relative ROS level. As shown in Figure 2F and 2G, the treatment of ADR resulted in the decrease of intracellular GSH accompanied with the increase of ROS level. Meanwhile, pretreatment with DMY could reverse the intracellular redox state triggered by single exposure of ADR, which further indicated the decreased ROS level in DMY+ADR group comparing with that in ADR mono-treated group, might be involved, at least partially, in the cardio-protective effect of DMY against ADR.

### Dihydromyricetin-driven cardioprotection is dependent on MDM2-mediated ubiquitylated degradation of ARC

**Figure 3:**
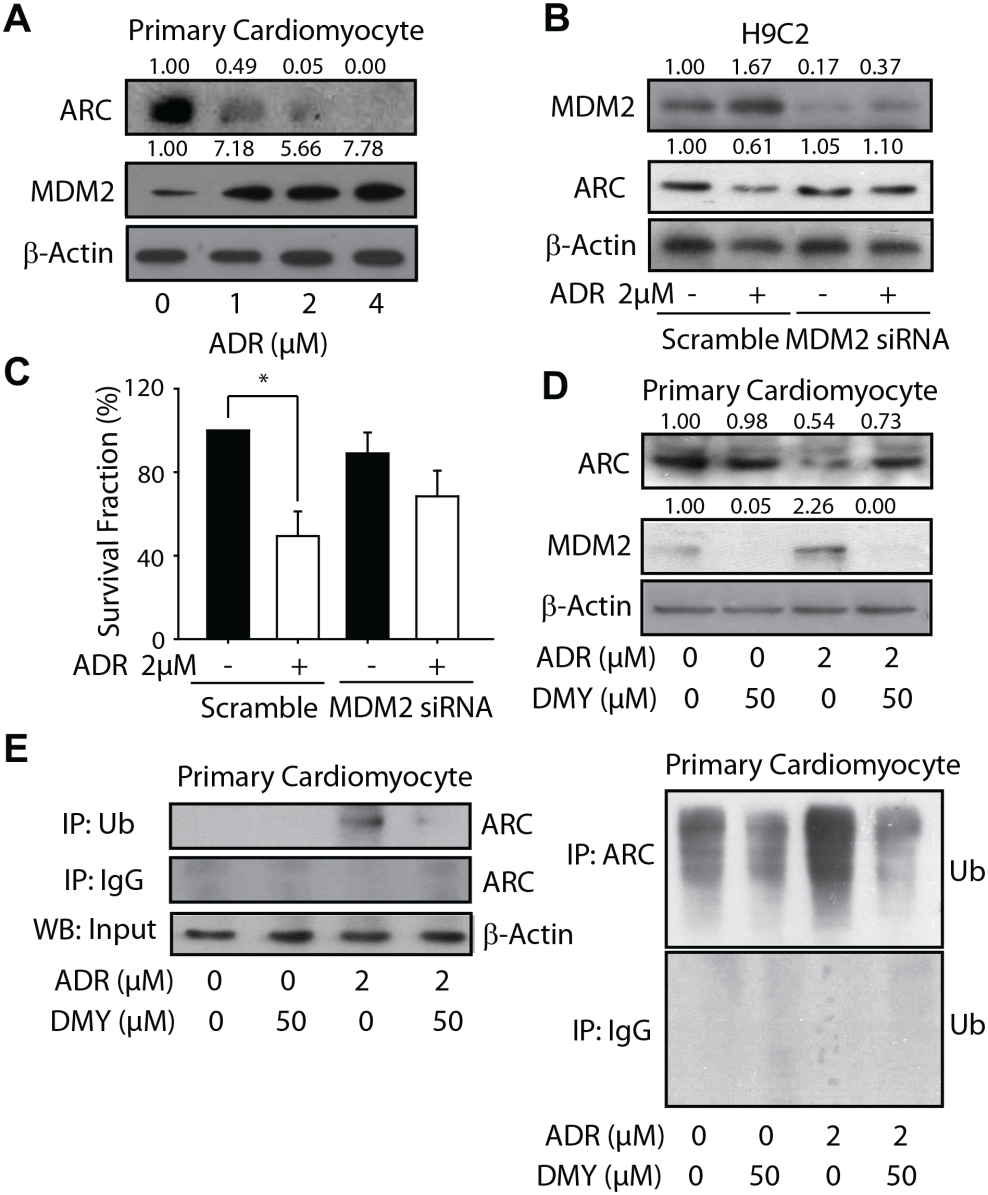
Dihydromyricetin accumulated ARC protein through restraining the MDM2-mediated ubiquitylated degradation **(A)** The influence of treatment of ADR on ARC and MDM2 expression. The neonatal rat heart myocytes were seeded into 6-well plates and treated with ADR in a series concentration for 24 h. ARC and MDM2 expression were analyzed by western blot. **(B and C)** MDM2 was involved in ADR-induced cardiotoxicity. The H9C2 cells were transfected with siRNA targeting MDM2 and seeded into 6-well plates and 96-well plates respectively, ADR of 2 μM was added and the expression of MDM2 and ARC were detected by western blot 24 h later (B) and the survival fraction was measured using MTT assay (n=4) (C). **(D and E)** The treatment of DMY accumulated ARC protein by inhibiting the ubiquitylated degradation mediated by MDM2. The primary cardiomyocytes were seeded into 6-well plates and treated with ADR (2 μM) or DMY (50 μM) or ADR (2 μM) with pretreatment of DMY (50 μM) for 24 h. ARC and MDM2 expression were analyzed by western blot (D). The effect of DMY on interaction relationship of ARC and ubiquitin were detected by immunoprecipitation (n=3) (E).

Although several lines of evidence indicate the critical roles of oxidative stress in ADR-induced cardiotoxicity, some inconsistent results were achieved when researchers employed antioxidants trying to prevent the cardiotoxicity [25]. In line with these evidences, our data reveal that, although both DMY and vitamin C exhibits the activity of scavenging the free radicals (Figure S2A), only DMY could protect the cells from the ADR-driven cytotoxicity (Figure S2B). Thus some mechanisms other than oxidative stress might be involved in ADR-induced cardiotoxicity. Given that recent evidence has emerged that ADR could directly interact with cell death pathways [26], the regulation of apoptotic factors might play critical roles in the cardiotoxicity. In an attempt to further explore the underlying molecular mechanism by which DMY to exert cardioprotective activity, we mainly focused on the ARC protein, because (i) our preliminary data suggested that DMY could attenuate ADR-induced apoptosis, which probably owing to modulation of apoptotic related factors, (ii) ARC is an endogenous inhibitor of apoptosis which could protect the cells from stress-induced cell death, (iii) ARC is enriched in terminally differentiated cells and is recognized as a heart specific protein [27] (Figure 2). As shown in Figure 3A and Figure S3A, ADR markedly down-regulated the protein expression of ARC in both primay cardiomyocytes and H9C2 cells. Since MDM2 is an E3 ubiquitin ligase of ARC, we also detected the MDM2 expression in ADR-treated cells and found an accompanying elevalation in MDM2 expression. Thus the inverse correlation between ARC and MDM2 upon ADR exposure implied that the ubiquitylated degradation of ARC might be involved as the crosscurrents of MDM2. To address this point, we firstly transfected H9C2 cells with specific siRNA targeting MDM2, and the results indicated that ADR failed to down-regulate the ARC protein level with diminished MDM2 expression (Figure 3B). Importantly, the cell proliferation assay suggested that the knockdown of MDM2 reversed the cytotoxicity caused by ADR in H9C2 cells (Figure 3C), suggesting the critical role of MDM2-ARC axis in ADR-induced cardiotoxicity.

In this context, it is intriguing to know whether the manipulation of MDM2-ARC axis by DMY contribute to its cardioprotective effects against ADR. We detected the expression of ARC and MDM2 when the primary cardiomyocytes and H9C2 cells exposed to ADR and DMY or the combination. The western blot analysis results showed that DMY could negatively regulate MDM2 protein level and blocked the descending expression of ARC mediated by ADR (Figure 3D and S3B). To further validate whether the modulation of MDM2 caused by DMY could affect the ubiquitination of ARC, co-immunoprecipitation was performed after administration of indicated compounds in primary cardiomyocytes. As illustrated in Figure 3E, ubiquitin tagged ARC was remarkably increased when the cells were exposed to ADR as a single agent while pretreatment with DMY could reduce the ubiquitination of ARC significantly. These data further implicated that DMY-driven cardioprotection was dependent on MDM2-mediated ubiquitylated degradation of ARC.

### Dihydromyricetin enhances the anti-tumor activity of adriamycin in a p53–dependent manner

**Figure 4:**
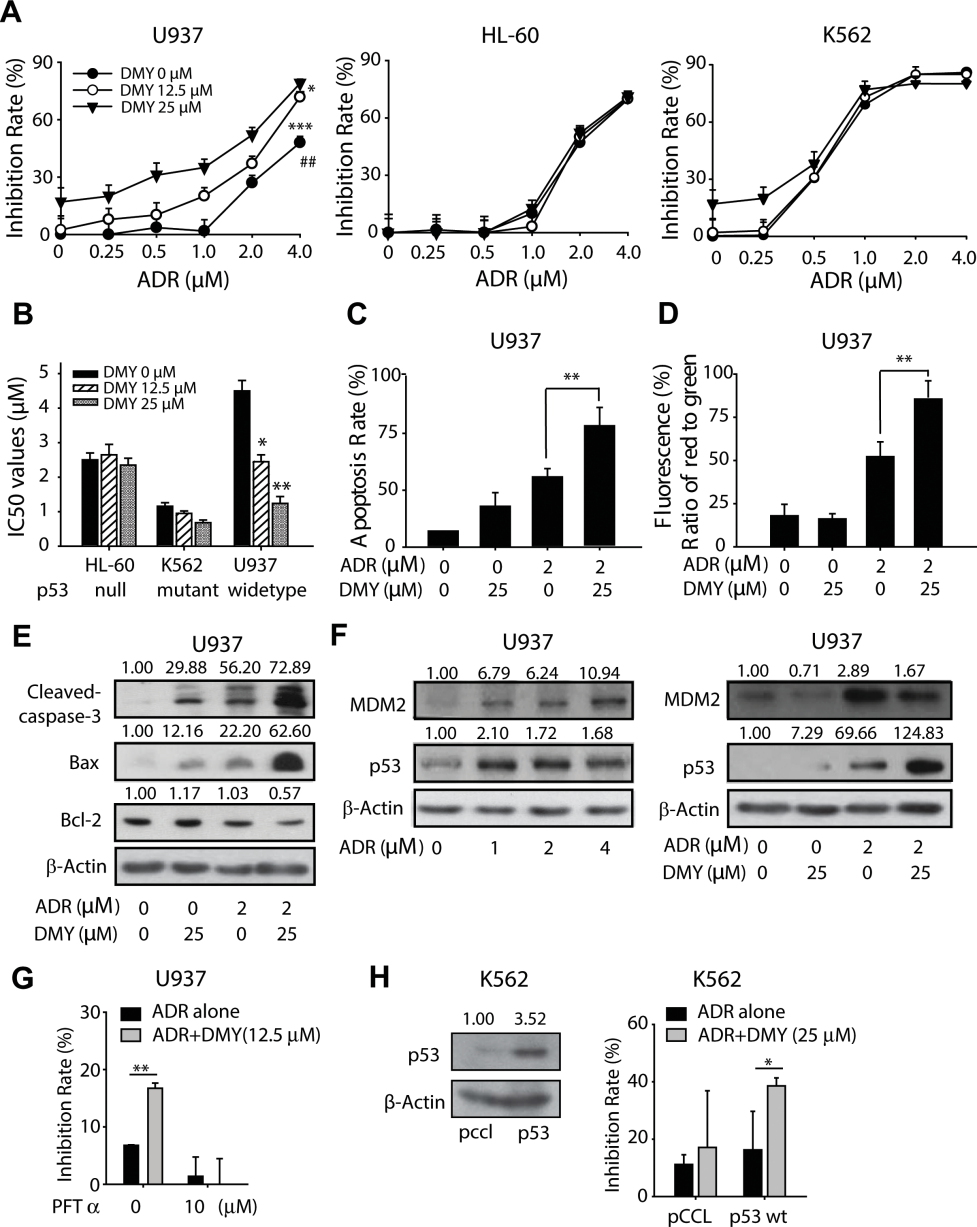
Dihydromyricetin sensibilized the anticancer activity of Adriamycin in p53 wild type cancer cells **(A and B)** Effects of DMY on ADR induced growth arrest in U937, HL-60 and K562 cells. P53 wild type cell line U937, p53 null cell line HL-60 and p53 mutant cell line K562 were seeded into 96-well plates, after pretreatment with DMY for 24 h, cells were exposed to ADR for another 24 h, the inhibition rate was detected using MTT assay (n=4) (A), and the IC50 was also valued (B). **(C)** Effects of DMY on apoptosis in U937 cells. Cells with the treatment of ADR (2 μM) or DMY (25 μM) as a single agent or co-administered agent for 24 h were harvest to analysis the apoptosis by flow cytometry using propidium iodide staining (n=3). **(D)** Effects of DMY on the loss of mitochondrial membrane potential in U937 cells. Cells were treated as described above and detected the loss of mitochondrial membrane potential by flow cytometry using JC-1 staining (n=3). **(E)** Effects of DMY on apoptosis-related protein expression in U937 cells. Cells were treated as described above and whole cell lysates were analyzed, cleaved-caspase3, Bax and Bcl-2 protein expression were analyzed by western blot. **(F)** The treatment of DMY accumulated p53 in U937 cells. P53 and MDM2 expression were analyzed after the treatment of ADR alone in a serious of concentration or combined with DMY. **(G)** p53 inhibitor PFT-α attenuated the cytotoxicity caused by DMY combined ADR in U937 cells (n=3). **(H)** K562 cells were transduced with lentiviral p53-pCCL or the empty vector. p53 overexpression increased the anti-cancer activity possessed by the combination of DMY and ADR in K562 cells. K562 cells transduced with lentiviral p53-pCCL or pCCL were exposed to DMY (25 μM), ADR (0.25 μM) and the combination for 24 h, and MTT assay was utilized to detect the cytotoxicity (n=3).

The search for cardioprotective agents that alleviate ADR cardiotoxicity in the last 40 years has led only to dexrazoxane. However, apart from the higher incidence of myelodysplastic syndrome reported in recent years, the negative effect on the antineoplastic activity possessed by ADR in clinical couldn't satisfied the medical researchers. Hence, it would be of great importance to evaluate the impact of cardioprotective agents on the anticancer activities of the chemotherapy. Consequently, cytotoxicity assay was employed using a variety of human leukemia cells as models. Of interest, in p53-null cell line HL-60 and p53-mutant cell line K562, the inhibition ratio of DMY+ADR groups remained unchanged, comparing to ADR mono-treated groups; in contrast, in p53-wild-type cell line U937, DMY augmented the cytotoxicity of ADR in a concentration-dependent manner (Figure 4A and 4B). In addition, we further determined the cytotoxicity when ADR was combined with dexrazoxane. As shown in Figure S4, the co-treatment with dexrazoxane attenuated the cytotoxicity of ADR (24 h). The inhibition ratio of ADR at the concentrations of 1, 2, and 4 μM was 29.7%, 54.1% and 67.1%, respectively; but decreased to 2.7%, 30.9% and 48.2% when combined with dexrazoxane. This result suggested that dexrazoxane impaired the anti-cancer capability of ADR in U937 cells, which could be owing to dexrazoxane-mediated interruption of ROS generation [28] and formation of topoisomerase II cleavage complexes caused by ADR [29].

Further studies were taken out to monitor the apoptotic population, the mitochondrial membrane potential and the apoptosis-related protein expression in U937 cells, upon the treatment of ADR with or without DMY. The results showed that when combined with DMY, ADR-induced apoptosis would be increased, as indicated by the enhanced apoptosis rate, decreased mitochondrial membrane potential and activated caspase cascades with up-regulated Bax and down-regulated Bcl-2 protein. These findings suggested that DMY could cooperate with ADR to increase the cytotoxicity effect in p53-wild-type cell line U937, and the possibility was raised that the functional p53 might be indispensable for the cooperative killing of cancer cells (Figure 4C, 4D and 4E). Consequently, we further examined the expression of p53 and MDM2 in U937 cells administrated with increasing concentration of ADR. Consistence with our data mentioned above, MDM2 was up-regulated in ADR-treated U937 cells (Figure 4F), accompanied with the induced expression of p53 along with the increasing concentration of ADR. Importantly, the pretreatment of DMY could decrease the MDM2 expression and accumulate p53 expression in combination with ADR (Figure 4F).

In addition, a p53 inhibitor Pifithrin-α (PFTα) was introduced [30], and the cytotoxicity elicited by DMY and ADR on U937 cells were determined, with or without the pre-treatment of PFTα for 24 h. As shown in Figure 4G, without pretreament of PFTα, DMY significantly enhanced the cytotoxicity in ADR-treated cells; whereas PFTα (10 μM) abolished the increased anti-cancer activity, with the cytotoxicity of the combination group reduced from 16.74% to 0.02%. Furthermore, a lentiviral plasmid encoding wide-type p53 (p53-pCCL) was used to reconstitute exogenous expression of p53 in K562 cells (Figure 4H). And the reconstitution of wide-type p53 could extend the cytotoxicity in DMY+ADR groups, as indicated by the increased inhibition ratio from 16.2% (ADR alone) to 38.5% (ADR+DMY) in p53 overexpressed K562 cells, which was more robust than that in those K562 cells without wide-type p53. Collectively, these data implied the critical role of the activated wide-type p53 in DMY-sensitized ADR exerting anticancer activity in human leukemia cells.

### Dihydromyricetin in combination with adriamycin possesses synergistic antitumor effect *in vivo*

**Figure 5:**
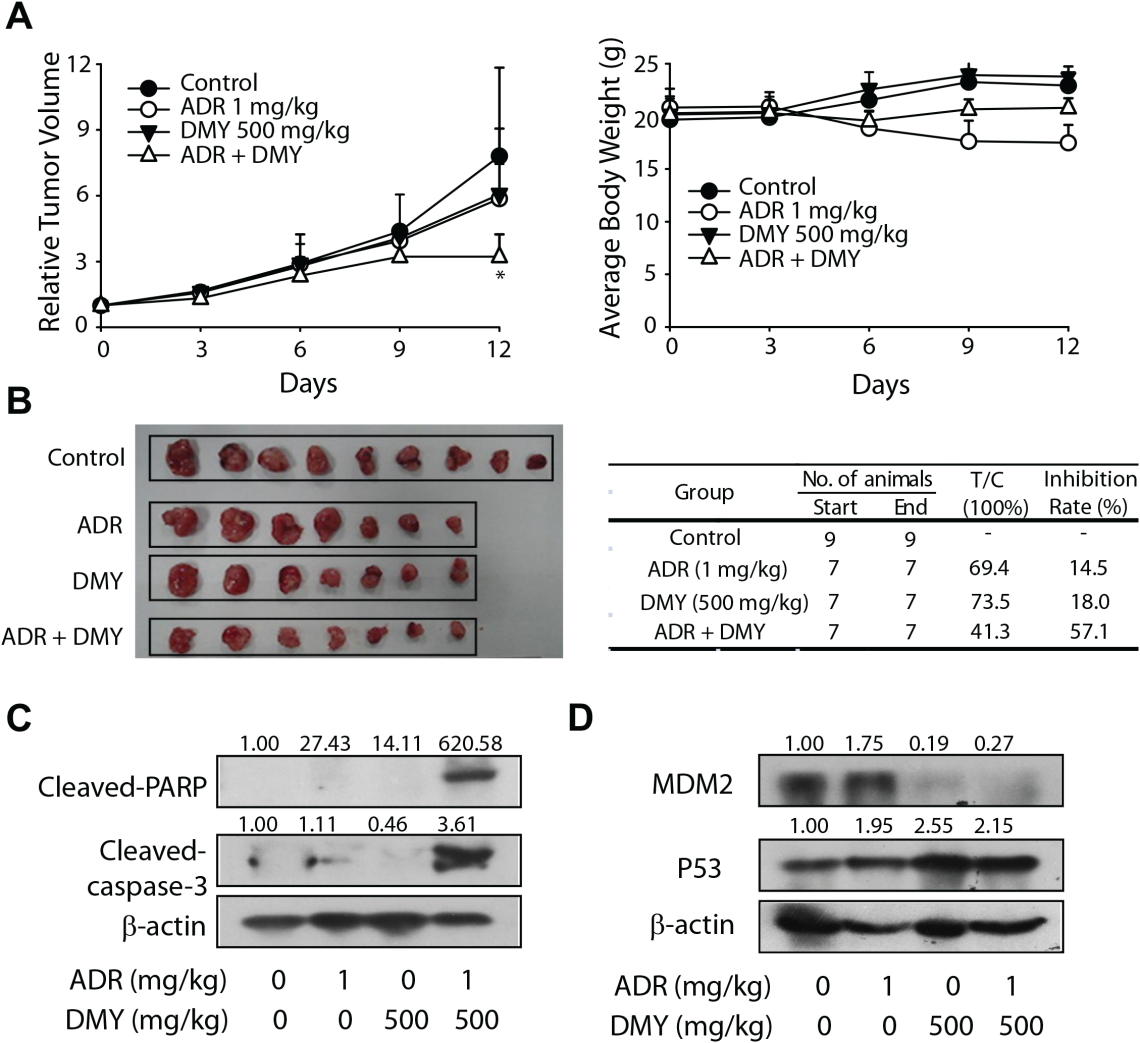
Dihydromyricetin possessed synergistic antitumor effect with adriamycin *in vivo* **(A)** The relative tumor volume and average body weight of BALB/C mice. ADR (ip) and DMY (ig) was administered two or three times a week. The tumor volume and the body weight were valued every day. **(B)** The picture of the tumor and the T/C value with inhibition rate. The solid tumors were removed and weighed on the twelfth day, the T/C value and the inhibition rate were also calculated. **(C)** The expression of cleaved-PARP and cleased-caspase3 in tumor tissue. **(D)** The expression of p53 and MDM2 in tumor tissue.

To further characterize the anticancer efficacy conducted by DMY combined with ADR, the nude mice human leukemic U937 xenograft models were introduced. DMY (ip) and ADR (ig) were administrated every day after the tumor volumes coming to about 300 mm^3^. As illustrated in Figure 5A and 5B, the inhibiting effect of ADR on the tumor growth as a single agent at the dose of 1 mg/kg was weak accompanied with a reduced body weight since day 6 from the drug administration. However, the simultaneous treatment of ADR and DMY increased the tumor growth inhibition rate from 14.5% to 57.1% with a decreased body weight loss at the same time. Western blot analysis of the proteins extracted from tumor tissues showed that there were a robustly increased cleavage of caspase3 and PARP when combined DMY with ADR, denoting the activation of caspase cascades (Figure 5C). And the subsequent results corroborated that MDM2 expression was down-regulated by DMY, and p53, as an important target on anticancer and antitumorigenesis, was accumulated (Figure 4D). Thus in addition to the findings achieved from *in vitro* models, our *in vivo* data also revealed that DMY in combination with ADR exhibited synergistic anticancer effect, particularly, in human leukemic cell models.

## DISCUSSION

Cardiotoxicity is a serious side effect of anthracycline chemotherapy, limiting the use of these most effective anticancer drugs, among which, ADR is one of the most widely used anthracycline antibiotic in a variety of cancer models. ADR was first discovered in the 1960s as a successful operative therapy option in treatment of acute leukemia and lymphoma. However, in the past 40 years the application of ADR was seriously limited due to its irreversible damage on myocardial cells. Although numerous attempts have been devoted, the mechanisms beneath ADR-associated cardiotoxicity have not been fully clarified. There are extensive literatures postulating various molecular mechanisms responsible for ADR-induced cardiotoxicity such as increased oxidative stress, direct interference with iron homeostasis, inhibition of DNA replication and protein synthesis [31, 32].

Among these, the oxidative stress and disruption of intracellular iron accumulation hypotheses have been regarded as the most widely-accepted mechanisms [33–36]. Although several lines of evidences reported that some antioxidants and iron chelators could exert cardioprotective effects against ADR provoked cardiotoxicity, some other studies yielded inconsistent results [37–41]. These negative results achieved from iron chelators not only reveal that the intracellular iron disruption might not be the only major causes for the cardiotoxity induced by ADR, but also question the theory that the iron-chelating activity is the mechanism whereby dexrazoxane, the current only FDA-approved drug for ADR caused cardiotoxicity, exerts its function. Collectively, we have reason to speculate that in addition to oxidative stresses and intracellular iron accumulation, more complicated mechanisms are involved in ADR-induced cardiotoxicity, thus the exploration of novel cardioprotective agents should not be limited to these two targets/signaling pathways.

As a manner of fact, despite of the multifactorial and complicated mechanisms underlying ADR-caused cardiotoxicity, the eventually apoptotic death of the cardiac myocytes would directly and irreversibly lead to the development of CHF [42]. In this context, the strategy which could prominently reduce ADR-triggered apoptotic cell death of myocardial cells should be able to prevent the loss of cardiac myocytes. And the manipulation on the apoptosis-related factors could be regarded as an efficient way to counteract against the cardiotoxicity caused by ADR thus maintaining the cardiac performance.

Among the anti-apoptotic factors, ARC is one of the most predominantly accumulated proteins in myocardial cells, skeletal myocytes and neurons, possessing the antagonistic property of both the extrinsic and intrinsic cell death pathways [43]. It has been revealed by several lines of evidences that ARC is a critical cardiomyocyte survival switch in ADR cardiotoxicity [44]. Mounting evidences suggest that the mechanism by which ARC exert the anti-apoptotic effects is achieved by its function to prevent the conformational activation and translation of Bax, a pro-apoptotic Bcl-2 family member. In response to ADR, Bax would translocate from cytosol to mitochondria, accompanied with the dissipation of the mitochondrial membrane potential, the releasing of cytochrome c and the activation of caspase cascades, which ultimately initiate the undergoing of apoptosis [45, 46]. In consistent with the previous reports, in the present study, a remarkable decreased ARC expression was observed when the cells were exposed to ADR, accompanied with the increased accumulation of Bax, which might be vital to the apoptotic cell death in primary myocardial cells. Intriguingly, this loss of ARC caused by ADR was significantly abrogated in DMY-pretreated primary cardiomyocyte and H9C2 cells, implicating the involvement of ARC levels rescued by DMY in the cardioprotective effects exerted by this agent.

ARC was reported undergoing degradation mediated by ubiquitin-proteasome systems in response to death stimuli [47]. This notion was validated in recent report revealing that ADR could enhance the UPS-mediated degradation of ARC, and the proteasomal inhibitor partially rescued both ADR-downregulated ARC and apoptosis induction. In some other reports, the ubiquitin E3 ligase MDM2 has been found to directly accelerate ARC protein turnover via ubiquitination and proteasomal-dependent degradation [48]. These clues prompted us to speculate that MDM2, the E3 ubiquitin ligase of ARC, not only played roles in the decreased level of ARC under the treatment of ADR, but also might be served as a critical factor being modulated to rescue the decline of ARC, so as to exert the cardioprotective effect. This hypothesis was confirmed by our further study. As demonstrated in Figure 3A, ADR robustly increased the MDM2 protein level in primary cardiocytes, which may resulted in the degradation of ARC as indicated by the accompanying reduction of ARC. However, when the cells were pretreated with DMY, the enhanced MDM2 was antagonized, consequently, the degradation of ARC was prevented and the protein was stabilized.

Nevertheless, in consideration of the different destination from cardioprotection to cytotoxicity in neoplastic cells, it is difficult but important to reserve the anticancer effect of ADR when the cardioprotective agents were used. About which, it is also one of the risks of applying dexrazoxane, that probably compromise the anticancer efficiency of ADR. In the present study, we uncovered that DMY could accumulate p53 in the cells with functional wide-type p53, and enhanced the antitumor activity of ADR accompanying with a decline in the protein level of its E3 ubiquitin ligase MDM2. As a crucial cell cycle regulator and tumor suppressor, p53 has been extensively studied. Being described as “the guardian of the genome”, p53 can activate DNA repair, arrest growth and initiate apoptosis in response to myriad stressors and contribute to anticancer activity both before and after tumorigenesis [49]. In our study, the increased anticancer activity of combining ADR and DMY were observed in U937 cell and xenograft models which harboring wide-type p53. It indicated that the enhanced cytotoxicity by DMY might depend on the functional wide-type p53. Beyond that, the further mechanism about how DMY influencing the p53 and ARC in the given organization was still unclarified. Even so, several lines of evidence indicate a negative modulation relationship between ARC and p53 [50]. It was found that the ARC promoter contains an optimal binding site for tumor suppressor p53, by which p53 negatively regulated the expression of ARC. In consistent with this finding, p53 was also found possessing the ability to antagonize the anti-apoptotic function of ARC in the cytosol by driving apoptosis directly. On the other hand, ARC displayed suppressive effect on p53 as well. Due to the shared E3 ubiquitin ligase MDM2, the regulatory interplay between ARC, p53 and MDM2 was tightly interlinked and complicated. In an attempt to examine the ARC protein level of H9C2 and U937 cells in parallel, we found that H9C2 cells harbored more abundant ARC than U937 cells did (Figure S4B). In addition, the ARC protein level could not be accumulated by DMY in U937 cells, neither with or without the cotreatement of ADR (Figure S4B). These observations not only implied the opposing effects of DMY might be attributable to the diverse accumulation of ARC in these different kinds of cells, but also supporting the notion that the regulatory mode of ARC is complicated, which has been shown to behave diversely in U937 cells and H9C2 cells.

It has been reported that DMY was capable to induce cell cycle arrest in osteosarcoma [51] and melanoma [52], which raise the possibility that the cell cycle arrest caused by DMY in rapid dividing cancer cells probably associated with the inhibitory effects in these cells. However, under our experimental conditions, little impact of DMY on cell cycle distribution on U937 and H9C2 cells was observed (data not shown), thus excluding the involvement of cell cycle regulation in the differential effects against leukemia cells and cardiomyocytes.

The “paradoxical” effects imposed by one drug combination in cancer cells and the other cells have be mentioned and studied previously [53–55]. Daosukho et al found that a HDAC inhibitor phenylbutyrate (PBA) protected the cardiac tissue from ADR-induced ultrastructual damages, via the modulation of MnSOD. Although PBA interferes with multiple cellular targets and signaling pathways, including HDAC, ER stress, and ammonia scavenging in urea cycle dysfunction, it seems that the cardioprotective effects of PBA is more likely mediated by HDAC inhibition [56]. Of note, another study unraveled the mechanism underlying HDAC inhibitor-mediated protection of neurons from DNA damage caused by topoisomerase inhibitors. In mouse primary cortical neurons, HDAC inhibition prevents DNA damage-induced neurodegeneration by modifying the acetylation of K382 and K381 of p53 and thus suppresses its transcriptional activation of PUMA gene [57]. Intriguingly, a paradoxical phenotype was observed in cancer cells: that is, although the same lysine sites on p53 were acetylated, PUMA gene was activated and apoptosis was induced in these cancer cells. Taken together, because the specific outcomes of HDAC inhibition are similar with that imposed by DMY, to further examine whether DMY interfere with the HDAC function and whether the acetylation pattern of p53 was modulated by DMY in leukemia and myocardial cells may be beneficial to fully understanding the dual modes of action by DMY.

In summary, we have shown for the first time that DMY, a natural product extracted from ampelopsis grossedentata, possessed the cardioprotective effect contrarious with ADR through restoring the expression of anti-apoptotic factor ARC in myocardial cells. In addition to that, the anticancer activity of ADR could be enhanced by DMY in p53 wild-type U937 cells. The dual-mode of DMY in ADR-driven cardiotoxicity and anticancer activity might be attributed to the regulation on MDM2. These finding thus favors DMY a potential cardioprotective drug candidate to alleviate the cardiotoxicity of ADR in clinical, and amplified the application of ADR in oncotherapy.

## METHODS

### Cell culture

Mouse cardiac myoblast cell line H9C2, Human leukemia cancer cell line U937, HL-60 and K-562 were purchased from the Institute of Cell Biology in Shanghai. The H9C2 cells were cultured in DMEM medium (GIBCO, Grand Island, USA) and the other cells were cultured in RPMI-1640 (GIBCO, Grand Island, USA), supplemented with penicillin (100 U/ml), streptomycin (100 U/ml) and 10% FBS (Hyclone, Logan, UT, USA). All the cells were cultured at 37°C with 5% CO2 in a humidified atmosphere.

### Animal treatment

Neonatal Sprague-Dawley rats, ICR mice and BALB/C mice were supplied by the Shanghai Laboratory Animal Center, Chinese Academy of Sciences and housed in a clean grade room at 21 ± 1°C and 60 ± 5% humidity, under a 12-h light/dark cycle. Rats were fed sterile tap water and chow diet ad libitum from Shanghai SLAC Laboratory Animal Co. Ltd.

### The ICR mice model of cardiac toxicity of Adriamycin and the measurement of ALT, LDH and CKMB leakage

The ICR mice survival experiment was conducted (see Supplementary material) and the serum cardiac enzyme activity of AST, LDH and CKMB were detected by full-automatic biochemical detect machine (see supplementary material).

### Electrocardiogram recording

Echocardiography was performed in mice from each group, mice were anesthetized (ip) with sodium pentobarbital (0.05 mg/g) and fixed in a supine position. Electrocardiograms were recorded using a direct ink-writing 3-channel Mingograph (Biopac Systems Inc., CA, USA).

### Neonatal rat cardiomyocytes preparation

Neonatal rat cardiomyocytes were prepared as described in supplementary material.

### Cytotoxicity assay

The cytotoxicity was determined using MTT assay (see supplementary material).

### DAPI staining assay

Neonatal rat cardiomyocytes were cultured in 24-well plates and the changes of nuclei was photographed after DAPI staining with fluorescence microscope (see supplementary material).

### Analysis of apoptosis by propidium iodide staining

Cells (4×10^5^/well) were seeded into 6-well plates and the apoptosis rate was analyzed after PI staining by FACS-Calibur cytometer (see supplementary material).

### Measurement of intracellular ROS

The neonatal rat cardiomyocytes were seeded into 24-well plates (5×10^4^/ml) and the intracellular ROS was measured after incubation with Carboxy-DCFDA by fluorescence spectrometer (see supplementary material).

### Measurement of intracellular GSH

The content of GSH was detected using an assay kits (Nanjing Jiancheng, China) according to the manufacturer's protocol [19] (see supplementary material).

### JC-1 stain for mitochondrial membrane potential (ΔΨm)

The neonatal rat cardiomyocytes were seeded into 6-well plates (5×10^4^/ml) and after incubated with JC-1, samples were analyzed by FACS Calibur (see supplementary material).

### Western blot analysis

The western blot analysis was conducted as described in supplementary material.

### RNA interference

SiRNA against MDM2 or a non-targeting control siRNA, complexed with oligofectamin (Invitrogen, Carlsbad, CA, USA) following the manufacturer' instructions, were applied to H9C2 cells to yield a final concentration of 100 nM. After 4–6 h of transfection, cells were cultured in fresh DMEM. The sense sequence of MDM2 siRNA was 5′-AAUGCCUCAAUUCACAUAGAUTT-3′.

### Immunoprecipitation

Immunoprecipitation was performed as described previously [20] (see supplementary material).

### Lentiviral transduction

Lentiviral transduction was performed as previously described [21] (see supplementary material).

### Xenografts of human leukemia cancer and drug treatment

The U937 xenograft mice were divided into different group randomly and the drug treatments initiated when the tumors volume came to 300 mm^3^. The inhibition rate was calculated as [(average tumor weight of vehicle group-average tumor weight of test group)/average tumor weight of vehicle group] × 100% (see supplementary material).

### Statistical analysis

All values were expressed as mean ± s.d. Differences in the cytotoxicity of DMY, ADR and the combination groups in cardiomyocytes (Figure 2A) and leukemia cells (Figure 4A), the in vivo anti-tumor activity (Figure 5A) were evaluated by two way ANOVA analyses. The unpaired two-sided Student's t-test was employed to analyze the rest of the data. For each analysis, three independent experiments were conducted to obtain the data. * and ^#^ indicates the values are significantly different than the control (*p<0.05, **p<0.01, ***p<0.001, ^##^p<0.01).

## SUPPLEMENTARY DATA MATERIALS AND METHODS AND FIGURES


