# Supplementary material for: Dihydromyricetin prevents cardiotoxicity and enhances anticancer activity induced by adriamycin
Source: Oncotarget. 2014 Sep 5;6(5):3254–67. doi: 10.18632/oncotarget.2410 (PMC4413651; doi:10.18632/oncotarget.2410)
Supplement: Supplementary file 1 [file oncotarget-06-3254-s001.pdf]

SUPPLEMENTARY DATA

MATERIALS AND METHODS

The ICR mice model of cardiac toxicity of Adriamycin and the measurement of ALT, LDH and CKMB leakage

The ICR mice were divided into vehicle-treated group (control, n=10) and 20 mg/kg ADR-treated group (n = 10), 20 mg/kg ADR and 125 mg/kg DMY-treated group (n = 10), 20 mg/kg ADR and 250 mg/kg DMY-treated group (n = 10), 20 mg/kg ADR and 500 mg/kg DMY-treated group (n = 10) randomly. The ICR mice were treated with DMY (ig) for 4 days, then, ADR were treated (ip) on the fourth day to make the acute cardiac injury. The body weight and the death rate of the mice were measured every day. Serum cardiac enzyme activity of AST, LDH and CKMB were detected in the sixth day after drawing blood through tail vein and determined by full-automatic biochemical detect machine (Cobas c 311, Roche Diagnostics GmbH, Germany) using specific detective kits.

Neonatal rat cardiomyocytes preparation

Neonatal rat cardiomyocytes were prepared from 2 to 3-day-old neonatal Sprague-Dawley rats. Rats were euthanized by cervical dislocation and the cardiac apexes were retained only. The obtained tissue were kept in ice-cold D-Hank's balanced salt solution without Ca^{2+} and Mg^{2+} ions, washed three times with the same solution and minced into small fragments. Then cardiac cells were dissociated with trypsin (0.06%, w/v) for three or four times and obtained by centrifuging the combined digestion. To exclude non-muscle cells, the isolated cells were first plated in tissue culture dishes at 37°C for 4 h, and non-adherent cells were collected then incubated with bromodeoxyuridine (BrdU, 0.1 mmol/L) in the medium for three days to inhibit fibroblast growth. The neonatal rat cardiomyocytes were routine cultured in media DMEM, changed every two or three days, supplemented with 10% fetal bovine serum (FBS), penicillin (100 U/ml) and streptomycin (100 U/ml). After additional 72 h incubation, the monolayer cells were used in the experiments.

Cytotoxicity assay

Cells were seeded into 96-well plates with an appropriate density. After pretreatment with varying concentrations of DMY for 24 h, cells were exposed to ADR for another 24 h and the proliferation inhibition rate were determined using MTT assay. MTT was added (20.0 µl/well) and the plates were incubated for 4 h at 37°C. The purple formazan crystals produced in cells were dissolved in 100 µl DMSO and read on an automated microplate spectrophotometer (ThermoMultiskan Spectrum, Thermo Electron Corporation, Vantaa, Finland) at 570 nm. The IC50 value was calculated using the PrismPad computer program (GraphPad Software Inc., CA, USA) with Microcomputers.

DAPI staining assay

Neonatal rat cardiomyocytes were cultured in 24-well plates and treated with different drugs for indicated time. Washed the cells twice with PBS and then incubated with 4′,6-diamidino-2-phenylindole (DAPI) which was diluted with 0.1% Triton X-100 for 5 min. Photographed the changes of nuclei with fluorescence microscope (DMI 4000 B, Leica, Germany).

Analysis of apoptosis by propidium iodide staining

Cells (4×10^5/well) were seeded into 6-well plates and exposed to ADR, DMY or the both for indicated time. Cells were then harvested and washed with PBS, fixed with pre-cooled 70% ethanol at 4°C for 30 min. Cell were resuspended in 500 µl PBS containing 50 lg RNaseA and 5 lg PI at room temperature 30 min later in dark. For each sample 2×10^4 cells were collected and analyzed by FACS-Calibur cytometer (Becton Dickinson, CA, USA).

DPPH radical scavenging

The DPPH radical scavenging capacity was detected as follows. The different concentration of DMY and vitamin C was prepared and a volume of 2µl of each sample was added to an ethanolic solution of DPPH (25 µg/ml) to a final volume of 200 µl for 30 min in 96-well plate. The decrease in absorbance at 517 nm was determined with an automated microplate spectrophotometer (ThermoMultiskan Spectrum, Thermo Electron Corporation, Vantaa, Finland). The degree of DPPH radical scavenging activity was calculated as follows: inhibition rate (%) = (A517blank-A517sample)/ A517blank ×100%.

Measurement of intracellular ROS

The neonatal rat cardiomyocytes were seeded into 24-well plates (5×10^4/ml) and incubated at 37°C for 24 h. After the drug treatment for 3 h, the level of intracellular ROS was measured using the oxidation sensitive fluorescent dye Carboxy-DCFDA. An increase in green fluorescence intensity is used to quantify the generation of intracellular ROS. Carboxy-DCFDA was added at a final

concentration of 15 μM to the cell suspension, dissociated by EDTA, the neonatal rat cardiomyocytes were incubated at 37°C for 30 min, washed with PBS, and measured immediately by fluorescence spectrometer (Becton Dickinson, CA, USA) using an argon laser at 488 nm and a 535 nm bandpass filter. The fold change of ROS level was calculated as fluorescence per μg protein compared with untreated cells.

Measurement of intracellular GSH

The neonatal rat cardiomyocytes treated with different drugs for 24 h were harvested, lysed and the supernatants were collected to detect the content of GSH using an assay kits (Nanjing Jiancheng, China) according to the manufacturer's protocol[16].

JC-1 stain for mitochondrial membrane potential (ΔΨm)

The neonatal rat cardiomyocytes were seeded into 6-well plates (5×10^4/ml) and incubated for 24 h, then the cells were exposed to different drugs for next 48 h and harvested, washed by PBS twice, then resuspended in 0.5 ml of complete medium containing 10 μg/ml JC-1 for 30 min at 37°C. JC-1 is a cationic dye that exhibits potential-dependent accumulation in mitochondria, indicated by a fluorescence emission shift from green (525 ± 10 nm) to red (610 ± 10 nm). Samples (1×10^4 cells/sample) were analyzed by FACS Calibur (Becton Dickinson, CA, USA).

Western blot analysis

The protein samples of the neonatal rat cardiomyocytes or tumor tissues were extracted in lysate buffer and the total protein concentration of whole cell lysates was determined using the Bradford method (Bio-Rad, Hercules, CA, USA). 40.0–60.0 μg of total protein was loaded per lane and fractionated on 10–15% tris-glycine precast gels, transferred to PVDF membrane (Millipore, Bedford, MA, USA). The membranes were blocked with 5% non-fat dry milk in 0.01 M Tris Buffered Saline with 0.1% Tween-20 (TBST) for 1 h. Subsequently, the membrane was incubated with primary antibodies (1:500) directed against target proteins overnight at 4°C and HRP-labeled secondary antibodies diluted at 1:5000 in TBST for 1 h. Proteins were visualized using ECL.

Immunoprecipitation

Immunoprecipitation was performed as described previously. Briefly, cells were harvested and lysed using universal lysis buffer and the protein concentration was measured using the Bradford method (Bio-Rad, Hercules, CA, USA). Aliquots (500 μg) of cellular proteins were precleared by adding 1.0 μg of the appropriate normal immunoglobulin G together with 40 μl of appropriate protein A+G-agarose conjugate (Santa Cruz Biotechnology, CA, USA) for 1 h at 4°C. The immunoprecipitations were performed with the appropriate antibody for 2 to 16 h at 4°C. Complexes bound to the protein A+G-agarose conjugate were washed five times with universal lysis buffer and separated by SDS-PAGE.

Lentiviral transduction

Lentiviral transduction was performed as previously described. K562 cells were cultured in PRMI-1640 medium then plated onto 6-well plates. After 12 hours of plating, transductions were performed with multiplicity of infection (MOI) of 1:10, with lentiviral p53-pCCL subcloned from pCMV6-p53 purchased from Origene (Rockville, MD) or the pCCL vector, in the presence of 6 μg/ml polybrene (Sigma-Aldrich). After 16 hs, cells were washed and cultured with fresh medium.

Xenografts of human leukemia cancer and drug treatment

The U937 cells were first harvested by trypsinization and washed two times with PBS and then injected in a total volume of 0.2 mL (5×10^7 cells/ml) into the armpit of the BALB/C mice. The BALB/C mice were divided into vehicle-treated group (control, n=9) and 2 mg/kg ADR-treated group (n=7), 50 mg/kg DMY-treated group (n=7), 2 mg/kg ADR combined with 50mg/kg DMY-treated group (n=7) randomly. The drug treatments were two or three times a week initiated when the tumors volume came to 300 mm^3. The tumor volume and body weight were recorded every day. Twelve days later, mice were euthanized by cervical dissociation and solid tumors were removed and weighed. The inhibition rate was calculated as [(average tumor weight of vehicle group-average tumor weight of test group)/average tumor weight of vehicle group] × 100%.

SUPPLEMENTARY FIGURES

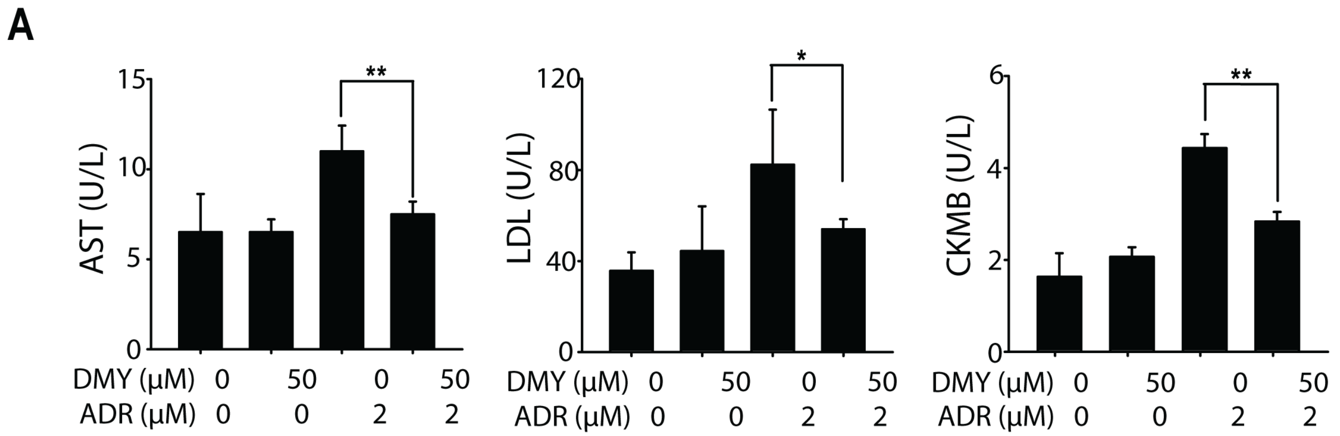

Supplementry Figure S1: Effects of Dihydromyricetin on Adriamycin induced release of intracellular AST, LDL and CKMB of cultured primary myocardial cells. (A) The influence of DMY on released AST, LDL and CKMB in cell supernatant caused by ADR. The primary myocardial cells were cultured with different drugs and the levels of myocardial enzymes AST, LDH and CKMB in cell supernatant were detected by full-automatic biochemical detect machine using specific detective kits. Data are expressed as mean±SD (n=4). *: $P < 0.05$; **: $P < 0.01$, compared with Adriamycin control. The results revealed that DMY could resist the increase of AST, LDL and CKMB level caused by ADR and decrease the myocardial injury.

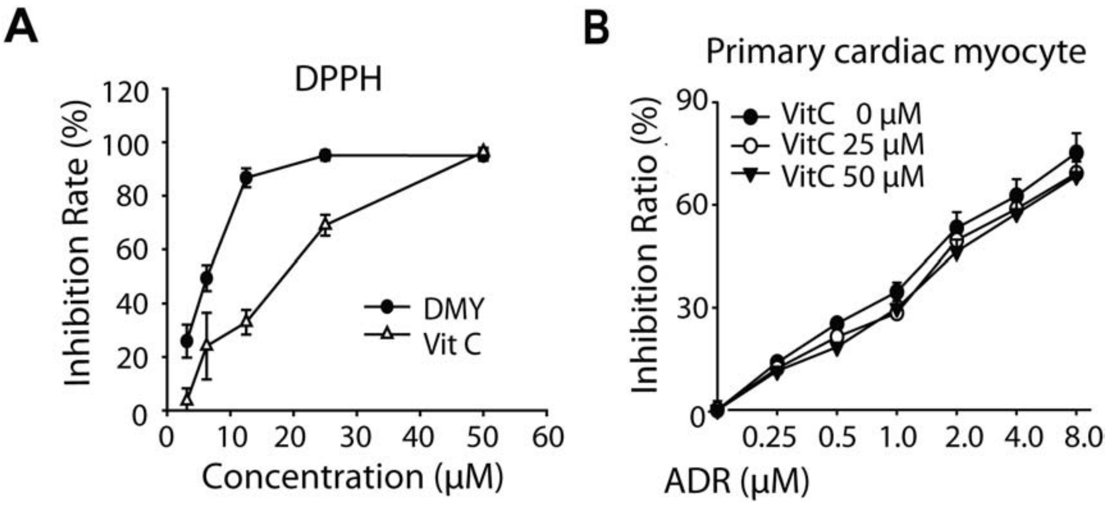

Supplementry Figure S2: The comparison of Dihydromyricetin and vitamin C on the capacity of scavenging the free radical and reversing the inhibition effect caused by Adriamycin on primary myocardial cells. (A) DPPH radical-scavenging activity of vitamin C and Dihydromyricetin. Data are expressed as mean±SD (n=4). **(B)** MTT Assay in detecting the inhibition rate of Adriamycin combined with Dihydromyricetin or vitamin C on primary cardiomyocytes. Cells were seeded at a density of 5×10^3 per well on 96 well plate and treated with DMY (DMY was pretreated 24 h earlier than ADR) and ADR as the indicated concentrations. MTT assay were conducted 24 h later after ADR treatment. Values were expressed as mean±SD (n=4). The Data here implied that scavenging the free radicals maybe not the efficient way and the core mechanism for DMY to possess the cardioprotective effect according to the comparison with vitamin C.

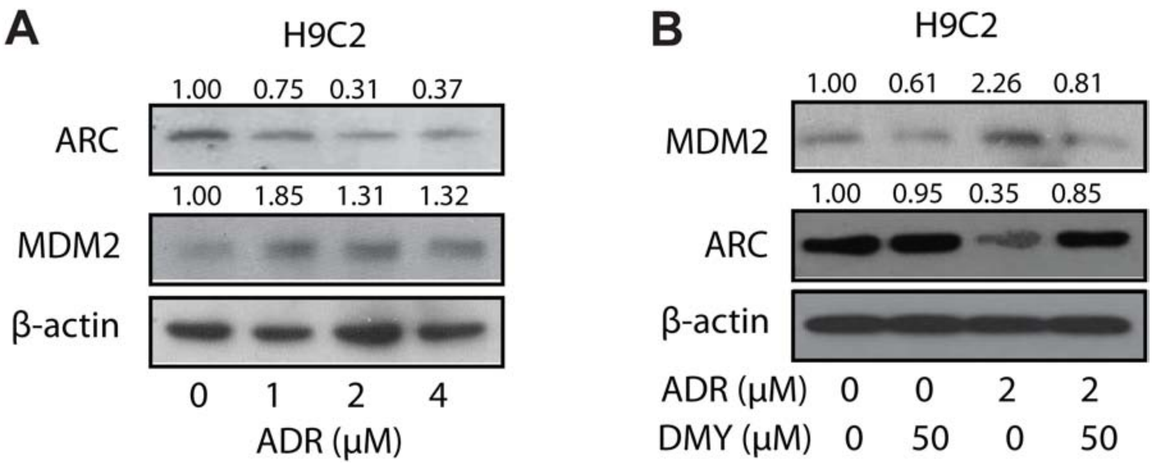

Supplementry Figure S3: Effects of Adriamycin and Dihydromyricetin on the expression of ARC and MDM2 in H9C2 cells. (A) The influence of treatment of ADR on ARC and MDM2 expression in H9C2 cells. The H9C2 cells were seeded into 6-well plates and treated with ADR in a series concentration for 48 h. ARC and MDM2 expression were analyzed by western blot and the results revealed that ADR could increase MDM2 expression and down-regulate ARC expression in H9C2 cells. **(B)** The influence of pretreatment of DMY with ADR on expression of ARC and MDM2. The H9C2 cells were seeded into 6-well plates (5×10^4/ml) and treated with ADR (2 μM) or DMY (50 μM) or ADR (2 μM) with pretreatment of DMY (50 μM, 24 h) for 48 h. ARC and MDM2 expression were analyzed by western blot and the data showed that DMY could accumulated ARC by inhibiting the expression of its E3 ubiquitin ligases MDM2 in H9C2 cells.

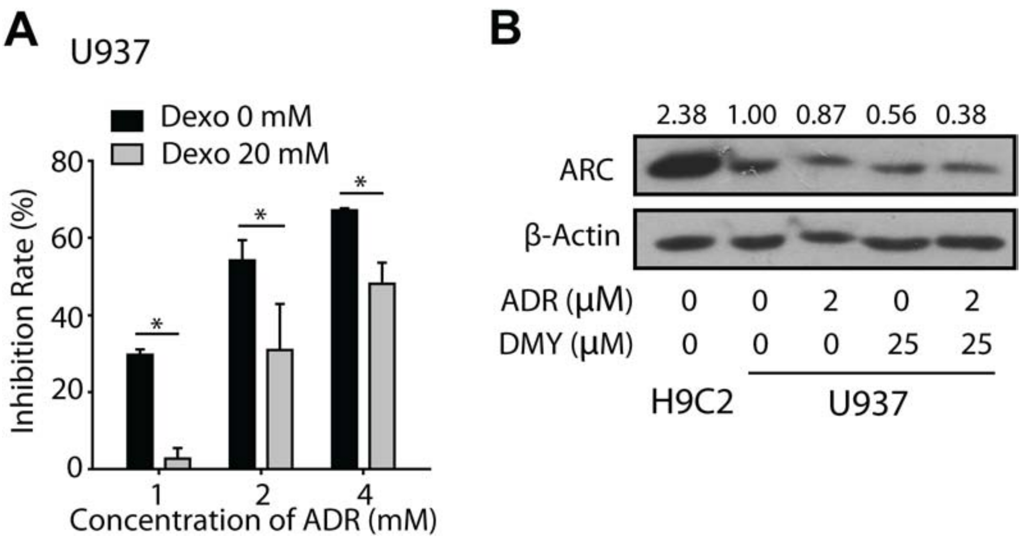

Supplementry Figure S4: Effects of dexrazoxane on ADR-induced cytotoxicity and the protein expression of ARC in H9C2 and U937 cells. (A) The cytotoxicity of combining dexrazoxane and ADR in U937 cells. The H9C2 cells were seeded into 96-well plates (5×10^4/ml) and treated with ADR in a series concentration (1, 2, 4 μM) for 24 h, with or without co-treatment of dexrazoxane (20 μM). MTT assay was utilized to determine the cytotoxicity (n=3). **(B)** The protein level of ARC in H9C2 and U937 cells. ARC expression were analyzed by western blot and the data showed that H9C2 harbored more abundant ARC protein than that in U937 cells. And DMY failed to accumulate ARC in U937 cells.
